# Sex-Specific Associations Between Serum Lipids, Antinuclear Antibodies, and Statin Use in National Health and Nutrition Examination Surveys 1999–2004

**DOI:** 10.3389/fmed.2022.887741

**Published:** 2022-05-26

**Authors:** Catherine J. Andersen, Terrence M. Vance

**Affiliations:** ^1^Department of Biology, Fairfield University, Fairfield, CT, United States; ^2^Department of Nutritional Sciences, University of Connecticut, Storrs, CT, United States; ^3^Department of Exercise and Nutrition Sciences, The State University of New York Plattsburgh, Plattsburgh, NY, United States

**Keywords:** serum lipids, cholesterol, statins, autoimmunity, NHANES, antinuclear antibodies (ANA), sex

## Abstract

Lipid metabolism contributes to the regulation of leukocyte activity and immune responses, and may serve as a therapeutic target in the pathophysiology and clinical management of autoimmune disorders. In addition to lipid-lowering properties, statins have been shown to exert anti-inflammatory and immunomodulatory effects within the context of autoimmunity. Importantly, autoimmune incidence and lipid markers differ between men and women, suggesting that the relationship between lipid metabolism and immune function may vary by sex. Therefore, we investigated whether a predictive, sex-specific relationship exists between serum lipids, statin use, and antinuclear antibodies (ANA)—a routine clinical marker of autoimmunity and immune dysfunction—in U.S. men and women (>20 years old; *n* = 1,526) from the National Health and Nutrition Examination Survey (NHANES) 1999–2004. Within this population, a greater proportion of women were positive for ANA (ANA+) and had higher ANA titers, as compared to men. While we did not observe statistical differences in average total cholesterol, LDL-cholesterol (LDL-C), HDL-cholesterol (HDL-C), or triglyceride levels in ANA positive (ANA+) vs. ANA negative (ANA–) men or women, we observed that a greater proportion of ANA+ women had high total cholesterol levels (>240 mg/dL) when compared to ANA+ men (13.0 vs. 9.0%), and that a greater percentage of ANA+ women had low HDL-C as compared to ANA+ men (29.2 vs. 19.6%). However, in logistic regression models, total cholesterol, LDL-C, and HDL-C levels were not able to predict ANA status, whereas elevated serum triglycerides (150 to < 200 mg/dL) were significantly less likely to be ANA+ vs. ANA– (OR 0.33; 95% CI 0.11–0.92) in men only. Interestingly, women who reported taking statins have significantly lower odds of being ANA+ (OR 0.25; 95% CI 0.09–0.76), whereas no significant association between statin use and ANA status was observed in men. Together, our findings provide novel insight into the relationship between lipid metabolism and autoimmunity by elucidating the limited, albeit sex-specific utility of routine clinical serum lipid levels to predict ANA status at the population level, while further identifying a sex-specific and protective role for statins in predicting ANA status in women.

## Introduction

Leukocyte selection, activation, and expansion is tightly regulated to ensure appropriate pathogen defense, immune surveillance of tumor cells, and resolution of inflammation to promote tissue healing and homeostasis (1–3). Compromised coordination of the mechanisms underlying immune responses can lead to impaired immunity, chronic inflammatory disorders, and autoimmune disease (4, 5). In autoimmune conditions, auto-reactive lymphocytes evade elimination during selection by lymphoid tissues and/or fail to be effectively regulated systemically, leading to the production of autoantibodies and inappropriate immune activation in response to self-antigens (6). Approximately 8% of the U.S. population −78% of whom are women—is affected by autoimmune diseases such as systemic lupus erythematous (SLE), multiple sclerosis, rheumatoid arthritis (RA), and type 1 diabetes mellitus, with the prevalence of many autoimmune conditions reported to be rapidly increasing (7–11). Autoimmune disorders are often complex to diagnose and treat, have a high economic burden, and can lead to chronic disability and death (8, 12). Apart from health complications directly associated with autoimmune conditions, SLE and RA patients—particularly premenopausal women—exhibit accelerated atherosclerosis and increased risk of cardiovascular morbidity and death (13, 14). Individuals with autoimmune disorders are similarly at increased risk for certain secondary autoimmune disorders, cancer, and infectious disease—including risk of COVID-19 (15–18). Thus, it is essential to develop effective prognostic tools and therapeutic strategies to minimize risk of developing advanced autoimmune complications (19).

Currently, antinuclear antibodies (ANA)—the most common type of autoantibodies—serve as routine clinical biomarkers in the diagnosis of autoimmune disorders (20). While a positive ANA status does not constitute a diagnosis of autoimmunity in the absence of additional measures, studies have demonstrated that the presence of ANA is evident in individuals who later develop autoimmune disease for years prior to the emergence of other clinical symptoms (21, 22). Accordingly, ANA positivity is associated with risk factors of autoimmunity, including smoking, age, infection, ethnicity, and sex (23–25). ANA positivity has additionally been shown to be more prevalent in lipid-related disorders and patients with severe coronary atherosclerosis as compared to healthy controls, and serves as a significant predictor of cardiovascular events and death (26, 27). ANA have additionally been identified in COVID-19 patients, with some reports indicating that patients who are positive for ANA have worse COVID-19 prognoses (28, 29). These findings have important implications for the use of ANA as a routine, preventative screening tool, and warrant the investigation of additional physiological parameters that influence ANA status to better improve the specificity and predictability of developing autoimmune disorders and associated comorbidities based on early ANA measures (30).

While the etiology of autoimmune disorders remains largely unknown, a growing body of evidence suggests that lipid metabolism plays an important role in regulating leukocyte activity and global immune responses, with implications for autoimmunity etiology, pathophysiology, and ANA status (5, 31). Animal studies have demonstrated that cholesterol-enriched atherogenic diets may induce and/or exasperate autoimmune-like conditions, whereas HDL-mediated efflux suppresses B cell expansion and autoantibody production (5, 32). Atherogenic lipid profiles have been associated with future RA development (33), while multiple studies have observed dyslipidemias and dysfunctional HDL in SLE, RA, and psoriasis patients (34–38). Further, lipid-lowering statins have anti-inflammatory effects in SLE and RA patients (39, 40), and may protect against RA development (41). These findings suggest that lipid metabolism is a promising target to mitigate autoimmune development and complications; however, the predictive relationship between clinical lipid and autoimmune biomarkers in the general population remains to be elucidated.

Therefore, we investigated the association between serum lipids, statin use, and ANA status in men and women from the National Health and Nutrition Examination Surveys (NHANES) 1999–2004, which is representative of the general U.S. population. Given the established differences in lipid metabolism (42, 43), ANA status (23, 24), and autoimmune incidence between men and women (11), we further evaluated whether the relationship between clinical lipid markers and ANA status was sex-specific, as this may help to better inform prognostic and therapeutic practices.

## Materials and Methods

### Study Design and Population

NHANES is an annual, cross-sectional survey of the non-institutionalized, civilian U.S. population conducted by the Center for Disease Control National Center for Health Sciences. This survey program collects data through interviews and physical examinations from ~5,000 individuals across 15 different U.S. states each year, and is considered to be nationally representative of the general U.S. population. Participant data from NHANES 1999–2000, 2001–2002, 2003–2004 were used for secondary data analysis in this study, which included adult men and women ≥20 years old. ANA data was available for a random subsample of participants within these survey cycles. The final sample size for our analyses consisted of 1,526 individuals, determined by the availability of data for all variables of interest. NHANES protocols for each survey cycle were approved by the National Center for Health Statistics Research Ethics Review Board. All participants provided informed consent. NHANES information, protocols, and datasets are available online at: https://www.cdc.gov/nchs/nhanes/.

### Collection of Biological Samples, Anthropometric Measurements, and Survey Data

Health assessments and collection of fasting blood samples were performed in NHANES mobile examination center laboratories. Blood samples were analyzed for fasting serum lipid levels and ANA positivity and titers. For serum lipids, blood samples collected in mobile examination center laboratories were processed and stored at −20 °C until shipment to the Johns Hopkins University Lipoprotein Analytical Laboratory for analysis. Serum cotinine—a biomarker of smoking status (44)—was additionally measured by isotope dilution-high performance liquid chromatography/atmospheric pressure chemical ionization tandem mass spectrometry (ID HPLC-APCI MS/MS). Body weight and height were measured to determine body mass index (BMI), whereas waist circumference was measured using a non-flexible tape. Participants provided self-reported data on education level, race/ethnicity, and statin usage to trained NHANES interviewers.

### Fasting Serum Lipids

Serum was isolated from fasting blood samples to determine clinical lipid profiles, including total cholesterol, LDL-cholesterol (LDL-C), HDL-cholesterol (HDL-C), and triglycerides. Enzymatic assays were used to directly measure serum total cholesterol and triglycerides, whereas serum HDL-C was measured via direct immunoassays or enzymatic assays following serum depletion of apolipoprotein B-containing lipoproteins using heparin-Mn^2+^ precipitation. LDL-C was estimated using the Friedewald equation: LDL-C (mg/dL) = total cholesterol (mg/dL) − HDL-C (mg/dL) − triglycerides (mg/dL)/5 (45). All serum lipid measures are expressed as mg/dL. To assess the distribution of serum lipid levels between ANA positive and ANA negative participants, the following clinically-relevant lipid categories were used: total cholesterol (Optimal: <200 mg/dL; Borderline High: 200 to <240 mg/dL; High: >240 mg/dL), LDL-C (Optimal: <100 mg/dL; Near/Above Optimal: 100 to <130 mg/dL; Borderline High: 130 to <160 mg/dL, High: 160 to <190 mg/dL, Very High: >190 mg/dL), HDL-C (High/Optimal: Men: ≥40 mg/dL, Women: ≥50 mg/dL; Low: Men: <40 mg/dL, Women: <50 mg/dL), and triglycerides (Optimal: <150 mg/dL; Borderline High: 150 to <200 mg/dL; High: ≥200 mg/dL) (43, 46).

### Antinuclear Antibodies

A subsample of stored serum samples collected in NHANES 1999–2004 surveys were assessed for immunoglobulin G (IgG) autoantibodies against human nuclear antigens. Autoantibodies were detected using a HEp-2 cell immunofluorescence assay from INOVA Diagnostics (San Diego, CA, USA). Staining intensity of autoantibodies was ranked from 0 to 4+, with samples undergoing staining analysis and interpretation by a minimum of two independent experienced evaluators (24). Samples were compared against autoimmune and healthy control reference serum samples. To ensure assay precision, analysis was automatically repeated for a minimum of every 50th sample. Repeated analysis was additionally performed for titering of all samples with staining intensity of 3+ and 4+, tittering of randomly selected samples, and samples where there were potential technical issues or discrepancy in interpretation between evaluators. Samples with staining intensities of 3+ and 4+ further underwent immunoprecipitation analysis for the presence of autoantibodies, in addition to autoantibody titer assessment by serial dilution to concentrations of 1:80, 1:160, 1:320, 1:640, and 1:1,280. Individuals were considered to be ANA positive (ANA+) with total ANA staining intensity of >3+ at each titer concentration (≥1:80), whereas individuals with total ANA staining intensity of <2+ were considered to be ANA negative (ANA–), in line with previous studies and reports from ANA reference laboratories (7, 24, 25).

### Statistical Analysis

All statistical analyses were performed using SAS version 9.4 (SAS Institute Inc., Cary, NC, USA). In order to account for the complex probability sample of NHANES, SAS SURVEY procedures and appropriate sample weights were used for all analyses. To determine sex effects in the relationship between ANA positivity and serum lipids, all analyses were performed separately for men and women. Descriptive statistics and distribution of serum lipids levels across groups were reported as counts and weighted percentages for categorical variables and means and standard errors for continuous variables. Differences in mean values for continuous variables were determined by one-way ANOVA and Tukey HSD *post-hoc* comparisons, whereas differences in counts for categorical variables were determined by Chi-square or Fisher's Exact tests. The association between blood lipid levels and ANA positivity was assessed using multiple logistic regression, with analyses adjusted for age, race/ethnicity, education, serum cotinine, BMI, waist circumference, statin use, and survey cycle. For logistic regression models of the association between statin use and ANA positivity, the results were reported for both minimally adjusted and fully adjusted analyses, with minimally adjusted analyses controlling for age and fully adjusted analyses controlling for age, race/ethnicity, education, serum cotinine, BMI, waist circumference, and survey cycle. For all analyses, a *P* < 0.05 was considered statistically significant.

## Results

### Descriptive Characteristics of Men and Women by ANA Status

Population characteristics according to sex and ANA status are presented in Table 1. The NHANES study population (*n* = 1,526) consisted of a greater number of women (*n* = 811, 53.1%) than men (*n* = 715, 46.9%). We additionally observed a slightly greater proportion of women who were ANA+ (17.4%) as compared to men (11.7%), in line with previous studies (24, 47). Accordingly, for those men and women who were ANA+, women tended to have higher antibody titers within higher sensitivity ranges for autoimmune disease diagnosis (48), as evidenced by a greater number of women having positive ANA titers of 1:320 or greater. We further evaluated parameters that are associated with increased incidence of autoimmunity and elevated ANA, including age, anthropometrics, smoking, and race/ethnicity (24, 49–51). Waist circumference did not significantly differ between men and women or by ANA status, whereas statistical trends were observed for age (*P* = 0.052) and BMI (*P* = 0.064), with ANA+ men trending toward a younger age, and ANA+ men and women trending toward a lower BMI. Regardless of ANA status, men had significantly greater mean serum cotinine levels as compared to ANA+ and ANA– women, suggesting a more prevalent smoking status among men, on average. Further, we did not observe overall sex-specific differences in the distribution of education level or race/ethnicity or across ANA+ and ANA– groups. While the overall study population, regardless of sex and ANA status, consisted of a larger proportion of White individuals (53.1%), followed by Hispanic (27.1%), Black (16.8%), and Other race/ethnicities (3.6%), it is important to note that Black men and women represented a greater proportion of ANA+ individuals as compared to ANA– groups (Black Men: 26.7% ANA+ vs. 16.1% ANA–; Black Women: 18.4% ANA+ vs. 15.8% ANA–), and that a greater percentage of Hispanic men were ANA– (14.5%) as compared to women (10.0%).

**Table 1:** Descriptive statistics of men and women in NHANES 1999–2004 (*n* = 1,526).

	**Men**				**Women**				
	**ANA+** **(*****n** **=*** **75)**		**ANA– (*****n** **=*** **640)**		**ANA+** **(*****n** **=*** **141)**		**ANA– (*****n** **=*** **670)**		***P-value***
	**Mean**	**SE**	**Mean**	**SE**	**Mean**	**SE**	**Mean**	**SE**	
**Age (years)**	42.1	2.5	45.3	0.8	48.7	1.6	46.8	0.7	0.052
**BMI (kg/m** ^**2**^ **)**	26.7	0.5	28.0	0.3	26.5	0.5	28.4	0.4	0.064
**Waist circumference (cm)**	96.1	1.5	99.6	0.6	90.6	1.5	94.0	0.9	0.694
**Serum cotinine (ng/mL)**	109.0^a^	23.4	76.6^a^	8.2	30.8^b^	12.1	47.1^b^	5.9	0.0004
	***n***	**%**	***n***	**%**	***n***	**%**	***n***	**%**	
**Race/Ethnicity**									
Black	20	16.3	103	9.1	26	13.2	106	10.3	0.304
Hispanic	17	10.0	179	14.5	37	11.8	178	10.0	
White	38	73.7	337	71.8	74	71.8	356	73.8	
Other	0	-	21	4.6	4	3.2	30	5.9	
**Education**									
<9th grade	15	8.8	100	7.1	14	3.4	110	6.7	0.589
9–11th grade	13	12.3	105	13.5	26	14.4	99	12.1	
High school	14	22.0	131	24.0	26	21.2	157	25.3	
Some college	18	30.6	170	29.2	44	32.1	180	31.4	
College graduate	15	26.4	134	26.2	31	28.9	124	24.5	
**ANA titer**									
1:80	0	0	N/A	N/A	0	0	N/A	N/A	<0.001
1:160	2	2.7	N/A	N/A	0	0	N/A	N/A
1:320	24	32.0	N/A	N/A	21	14.9	N/A	N/A
1:640	29	38.7	N/A	N/A	36	25.5	N/A	N/A
1:1280	20	26.7	N/A	N/A	84	59.6	N/A	N/A

*ANA, antinuclear antibody; BMI, body mass index; M, men; NHANES, National Health and Nutrition Examination Survey; SE, standard error; W, women. Differences in mean values for each parameter was determined by one-way ANOVA and Tukey HSD post-hoc comparisons, where values with different superscript letters (a, b) represent statistically different comparisons (P < 0.05). Chi-square or Fisher's Exact tests were used to compare differences in distribution of ethnicity, education, and ANA titer levels across groups. Reported percentages are weighted to account for the complex probability design of the survey*.

### Serum Lipid Profiles of Men and Women by ANA Status

We further evaluated the distribution of serum lipid levels across clinical ranges by sex and ANA status to determine whether trends exist between markers of autoimmunity and dyslipidemias. In comparing average serum lipid levels, there were few differences between ANA+ vs. ANA– men and women (Table 2). Total serum cholesterol, LDL-C, and triglyceride levels were similar between men and women, regardless of ANA status. As expected, HDL-C levels were higher in women as compared to men in both ANA+ and ANA– groups; however, differences in HDL-C between ANA+ vs. ANA– men (*P* = 0.074) and ANA+ vs. ANA– women (*P* = 0.383) were not significant. When comparing the distribution of clinical lipid ranges by sex and ANA status, we observed significant differences in total cholesterol and HDL-C, in addition to a statistical trend in LDL-C distributions (*P* = 0.068) and no group differences in clinical triglyceride range distributions. The majority of men and women—regardless of ANA status—had optimal total cholesterol levels (<200 mg/dL), and a greater proportion of ANA+ women had high total cholesterol levels (>240 mg/dL) when compared to ANA+ men (13.0 vs. 9.0%). Further, a greater percentage of women had low HDL-C as compared to men—regardless of ANA group. ANA+ women had low HDL-C as compared to ANA+ men (29.2 vs. 19.6%), as did ANA– women vs. ANA– (35.8 vs. 27.6%). We additionally observed that a smaller proportion of ANA+ men and women were taking statins compared to ANA– groups, with a greater proportion of ANA+ men reporting statin use compared to ANA+ women.

**Table 2:** Distribution of serum lipids in ANA+ and ANA– men and women in NHANES 19992004 (*n* = 1,526).

	**Men**				**Women**			
	**ANA+** **(*****n** **=*** **75)**		**ANA**– **(*****n** **=*** **640)**		**ANA+** **(*****n** **=*** **141)**		**ANA**– **(*****n** **=*** **670)**		***P***
	**Mean**	**SE**	**Mean**	**SE**	**Mean**	**SE**	**Mean**	**SE**	
**Total cholesterol (mg/dL)**	196.0	5.5	202.3	1.8	202.9	4.8	205.7	2.0	0.3302
**LDL-cholesterol (mg/dL)**	120.1	4.5	127.3	1.6	117.7	4.3	120.7	2.1	0.1752
**HDL-cholesterol (mg/dL)**	50.5^a^	1.7	47.2^a^	0.6	60.1^b^	1.8	57.9^b^	1.1	<0.0001
**Triglycerides (mg/dL)**	127.1	11.2	138.9	3.6	125.8	6.8	135.0	3.2	0.3269
	***n***	**%**	***n***	**%**	***n***	**%**	***n***	**%**	
**Total cholesterol (mg/dL)**									
<200 mg/dL	34	49.5	318	48.8	67	52.3	301	47.2	0.002
200 to <240 mg/dL	34	41.5	235	36.2	49	34.7	223	35.2	
>240 mg/dL	7	9.0	87	15.0	25	13.0	146	17.6	
**LDL-cholesterol (mg/dL)**									
<100	19	25.8	156	23.3	38	31.3	196	29.9	0.068
100 to <130	26	32.0	208	32.4	49	32.5	214	33.6	
130 to <160	23	33.2	170	27.2	36	27.1	169	23.5	
160 to <190	4	6.2	83	13.5	8	5.8	56	8.2	
>190	3	2.8	23	3.6	10	3.3	35	4.7	
**HDL-cholesterol (mg/dL)**									
M: ≥40; W: ≥50	58	80.4	472	72.4	102	70.8	435	64.2	0.002
M: <40; W: <50	17	19.6	168	27.6	39	29.2	235	35.8	
**Triglycerides (mg/dL)**									
<150	55	76.3	420	65.5	100	72.0	415	66.2	0.160
150 to <200	7	6.1	112	17.2	18	13.0	122	17.7	
≥200	13	17.6	108	17.4	23	15.0	133	16.0	
≥500	0	-	0	-	0	-	0	-	
**Statin use**									
No	65	92.5	566	89.5	136	96.5	605	90.3	0.027
Yes	10	7.5	74	10.5	5	3.5	65	9.7	

*ANA, antinuclear antibody; ANA+, ANA positive; CI, confidence interval; M, men; NHANES, National Health and Nutrition Examination Survey; OR, odds ratio; SE, standard error; W, women. Data are presented as mean ± SEM (mg/dL) for ANA+ men (n = 75) and women (n = 141), and ANA– men (n = 640) and women (n = 670). Differences in mean values for each parameter was determined by one-way ANOVA and Tukey HSD post-hoc comparisons, where values with different superscript letters (a, b) represent statistically different comparisons (P < 0.05). Chi-square or Fisher's Exact tests were used to compare differences in distribution of ethnicity, education, and ANA titer levels across groups. Reported percentages are weighted to account for the complex probability design of the survey*.

### Serum Lipid Profiles Predict ANA Status to a Limited Extent in Men

We next set out to determine whether serum lipid markers could predict the odds of testing positive for ANA using logistic regression, and whether the predictive potential of lipid markers varied between men and women. After adjusting for age, race/ethnicity, education, serum cotinine, BMI, waist circumference, statin use, and survey cycle, the odds ratios (OR) for ANA positivity were not impacted by total cholesterol, LDL-C, or HDL-C levels in either men or women (Table 3). Conversely, we observed a 67% lower odds of ANA positivity in men with elevated triglycerides (150 to <200 mg/dL), whereas this relationship was not observed in men with optimal (<150 mg/dL) or high (≥200 mg/dL) triglycerides, nor was there an association between triglycerides and ANA positivity in women.

**Table 3:** Association between blood lipids and ANA positivity among adults ≥20 years of age in NHANES 1999–2004 (*n* = 1,526).

	**Men**			**Women**		
	***n* (%) ANA+**	**OR**	**95% CI**	***n* (%) ANA+**	**OR**	**95% CI**
**Total cholesterol (mg/dL)**						
<200	34 (9.7)	1.00	(referent)	67 (18.2)	1.00	(referent)
200 to <240	34 (12.6)	1.18	(0.58, 2.40)	49 (18.0)	0.91	(0.47, 1.75)
≥240	7 (7.5)	0.61	(0.20, 1.89)	25 (14.6)	0.57	(0.28, 1.16)
**LDL-cholesterol (mg/dL)**						
<100	19 (10.9)	1.00	(referent)	38 (16.2)	1.00	(referent)
100 to <130	26 (11.1)	1.02	(0.44, 2.37)	49 (18.6)	1.10	(0.58, 2.09)
130 to <160	23 (11.9)	1.36	(0.66, 2.84)	36 (17.6)	1.18	(0.58, 2.41)
160 to <190	4 (4.6)	0.48	(0.13, 1.75)	8 (12.5)	0.73	(0.29, 1.88)
≥190	3 (11.5)	0.89	(0.17, 4.79)	10 (22.2)	0.68	(0.22, 2.10)
**HDL-cholesterol (mg/dL)**						
Men: <40; Women <50	17 (9.2)	1.00	(referent)	39 (14.2)	1.00	(referent)
Men: ≥ 40; Women ≥ 50	58 (10.9)	1.30	(0.51, 3.27)	102 (19.0)	1.03	(0.63, 1.70)
**Triglycerides (mg/dL)**							
<150	55 (11.6)	1.00	(referent)	100 (19.4)	1.00	(referent)
150 to <200	7 (5.9)	0.33	(0.11, 0.92)	18 (12.9)	0.81	(0.81, 1.78)
≥200	13 (10.7)	0.90	(0.35, 2.32)	23 (14.7)	1.13	(0.61, 2.09)

*ANA, antinuclear antibody; ANA+, ANA positive; CI, confidence interval; OR, odds ratio; NHANES, National Health and Nutrition Examination Survey. Data were analyzed using logistic regression adjusted for age, race/ethnicity, education, serum cotinine, BMI, waist circumference, statin use, and survey cycle*.

### Statin Use Predicts ANA Status in Women, but Not Men

Given the anti-inflammatory and protective effects of statins in autoimmunity (39–41), we evaluated whether statin use impacts the odds of testing ANA+ in a general population. Interestingly, we found that women who reported taking statins had significantly lower odds of being ANA+ as compared to women who did not take statins in both age-adjusted (71% reduced risk) and fully-adjusted (75% reduced risk) models (Table 4). This observation was sex-dependent, as no significant associations between statin use and ANA prevalence was identified in men.

**Table 4:** Odds of ANA positivity among adult (≥20 years of age) statin users vs. non-users in NHANES 1999–2004 (*n* = 1,526).

	**Men**		**Women**	
	**OR**	**95% CI**	**OR**	**95% CI**
Age adjusted	0.87	(0.32, 2.37)	0.29	(0.10, 0.83)
Fully adjusted	0.81	(0.27, 2.46)	0.25	(0.09, 0.76)

*CI, confidence interval; OR, odds ratio. From logistic regression adjusted for age, ethnicity, education, serum cotinine, BMI, waist circumference, and survey cycle*.

## Discussion

Lipid metabolism plays a significant role in regulating leukocyte activation and immune responses, which may have important implications in the pathophysiology and treatment of autoimmune diseases (5, 32, 40). Given the well-established sex differences in lipid metabolism, ANA status, and autoimmune disease risk (24, 52), as well as the increased risk of cardiovascular events and death in ANA positive individuals (26, 27), it is essential to evaluate the relationship between serum lipids and autoimmune markers in men vs. women, as this may inform risk assessment and therapeutic interventions. Further, it is important to determine whether routine clinical biomarkers have predictive utility in evaluating the lipid-autoimmune relationship, which has yet to be investigated at the population level. In this study, we observed sex-specific differences in the distribution of total cholesterol and HDL-cholesterol across clinical ranges between ANA+ and ANA– individuals, but that serum lipids have limited, albeit sex-specific, capacity to predict ANA status. Importantly, statin usage significantly reduced the odds of being ANA+ in women only. Together, these findings provide valuable insight into the relationship between lipid metabolism and autoimmunity at the population level by highlighting the limitations and sex-specific conditions of utilizing routine clinical biomarkers to predict ANA status. Moreover, we identified a clear sex-specific and protective role for statins in predicting ANA status, which warrants further investigation to evaluate the potential clinical and therapeutic implications.

Differences in ANA status and autoimmune prevalence between men and women are well-documented (23, 53). Approximately 78% of the 23.5 million individuals in the United States with autoimmune disorders are women, while ANA have been reported to be more prevalent in women in the general population as compared to men (11, 24). Our findings are consistent with these previous studies, in that we observed a greater proportion of women who were ANA+ (17.4%) as compared to men (11.7%). We additionally found that ANA+ women had higher ANA titers as compared to men, each having ANA titers of 1:320 or greater. While ANA can be detected in healthy populations as a result of exposure to pathogens or self-antigens during cellular apoptosis and turnover (54)—including SARS-CoV2 infection (28), ANA titers of 1:160 or greater may have a higher specificity and sensitivity in the diagnosis of autoimmune disorders (48). Differences in autoimmune disease risk between men and women may be attributable to sex hormone-mediated effects on immune cells, as well as differences in the regulation of acute and chronic immune responses (11). Estrogen receptor α (ERα) has been implicated in the suppression of T regulatory (T_reg_) cells, which protect against autoimmune dysfunction and pro-inflammatory T helper 1 (T_H_1), T_H_2, and T_H_17-mediated responses (55, 56). Females additionally display greater antibody production and T_H_2 cell-biased responses to infection and vaccination, whereas T_H_1 cell-biased responses are observed in males (57). Accordingly, autoimmune disorders that disproportionately affect women are associated with high autoantibody levels, as well as antibody-mediated and chronic pathologies (11). Thus, it is essential to consider sex as a significant confounding factor when investigating immune function and pathologies (58).

In evaluating the relationship between serum lipids and ANA status in men and women, we controlled for additional factors that are associated with ANA status and autoimmune risk, including age, BMI, smoking status, and race/ethnicity (24, 49, 59). In evaluating a subset of 4,754 individuals (≥12 years old) in NHANES 1999–2004, Satoh et al. (24) found that the ANA prevalence increased with age, and that African Americans were more likely to be ANA+ compared to White individuals. Increased risk of autoimmunity with aging is thought to be attributable to thymic involution and immunosenescence of T and B cells (60, 61), whereas the underlying cause of autoimmune disparities across racial/ethnic groups may be attributable to genetic and environmental factors (62). Interestingly, there was trend toward ANA+ men having a lower mean age, which corresponds with recent reports that immune aging is accelerated to a greater extent in men (63, 64). Further, while overall sex-specific differences in the distribution of race/ethnicity or education level across ANA+ and ANA– groups did not reach statistical significance, it is important to note that Black men and women represented a greater proportion of ANA+ individuals as compared to ANA– groups (Men: 26.7% ANA+ vs. 16.1% ANA–; Women: 18.4% ANA+ vs. 15.8% ANA–), and that a greater percentage of Hispanic men were ANA– (14.5%) as compared to women (10.0%), suggesting that sex may differentially impact autoimmune risk across racial/ethnic groups. We further observed differences in smoking status between ANA and sex groups, where men had significantly greater mean serum cotinine levels as compared to women, regardless of ANA status. While a study by Young et al. (65) did not find a relationship between cigarette smoking and ANA prevalence, smoking has been associated with a greater risk of developing SLE and RA (50, 66, 67). Obesity has additionally been linked to immune dysfunction and autoimmune disease (4, 68, 69), yet we did not observe significant statistical differences in mean BMI between ANA+ and ANA– groups for men or women.

Similar to ANA status and autoimmune disease risk, sex differences have been observed in various aspects of lipid metabolism (11, 23, 42, 70). Men are typically reported to have higher total cholesterol, LDL-C, and triglycerides as compared to premenopausal women, whereas lipid levels tend to equalize in men and postmenopausal women, which correspond to sex-specific differences in cardiovascular disease risk (70–72). In addition to having higher HDL-C as compared to men, women exhibit distinct HDL particle profiles and patterns of HDL-medicated cholesterol efflux (42, 43). Although the etiology underlying sex-specific variations in lipid markers remains to be elucidated, differences in sex hormones, body composition, and metabolism of fatty acids and complex lipids are thought to be involved (71, 73–75). Consistent with these studies, we observed significantly higher HDL-C levels in women as compared to men that was independent of ANA status, while we did not observe differences in total cholesterol, LDL-C, or triglyceride levels between men and women.

Evidence from animal studies and clinical trials demonstrates that lipid metabolism plays a direct and significant role in autoimmune disease risk, development, and treatment, suggesting that sex-specific lipid profiles may contribute to the sexual dimorphism of autoimmunity and subsequent risk of cardiovascular disease (5, 14, 27, 32, 52). Cell and animal-based studies demonstrate that cellular cholesterol loading is associated with serum hypercholesterolemia and a lower threshold to pro-inflammatory T cell activation, whereas lipid efflux via HDL-mediated efflux suppresses T and B cell expansion, inflammation, and autoantibody production (5, 32, 76, 77). Accordingly, we have recently demonstrated that serum lipids can differentially predict leukocyte subset counts in NHANES 1999–2004 in a sex-dependent manner (52), whereas various other population-based studies utilizing different cohorts and datasets have similarly identified predictive relationships between serum lipids and apolipoproteins vs. leukocytes counts (78–80). Further, atherogenic dyslipidemias and dysfunctional HDL are observed in SLE, RA, and psoriasis patients (34–38). In this current study, we observed that a greater proportion of ANA+ women had high total cholesterol levels (>240 mg/dL) when compared to ANA+ men (13.0 vs. 9.0%). We additionally observed that a greater percentage of ANA+ women had low HDL-C as compared to ANA+ men (29.2 vs. 19.6%), although differences in HDL-C between ANA+ vs. ANA– status in men and women did not reach significance. Despite variability in clinical lipid ranges between ANA+ and ANA– men and women, total cholesterol, LDL-C, and HDL-C levels did not predict ANA status in adjusted models. While HDL is known to mediate autoimmune dysfunction and inflammation (5, 32), immunomodulatory properties of HDL are most often attributable to functional components of the HDL particles (e.g., lipidome, proteome, and cholesterol-accepting capacity) that are independent of HDL-C levels (5, 81–83), which could explain why stronger associations between HDL-C and ANA status were not observed. Thus, further research is warranted to evaluate the extent to which degrees of hypercholesterolemia and HDL function may influence leukocyte cholesterol metabolism, activity, and autoantibody production in human populations, and whether additional routine clinical markers could be utilized to develop stronger predictive models.

Despite a lack of predictive associations between serum cholesterol markers and ANA status, we found that elevated serum triglycerides levels (150 to <200 mg/dL) were associated with a reduced odds of being ANA positive—an association that was only found in men, and not women. Hypertriglyceridemia is a result of increased lipogenesis in the liver and reduced systemic lipolysis, leading to greater secretion and circulation of triglyceride-rich very low-density lipoproteins (VLDL), respectively (84, 85). Intriguingly, our findings are in contrast with previous studies which have shown that elevated serum triglycerides can promote deposition of lipids in metabolic and lymphoid tissues, inducing direct activation of leukocytes by fatty acids, in addition to indirect immune activation in response to tissue dysfunction and inflammation that may exasperate autoimmune dysfunction (4, 86–88). We additionally did not observe a dose-dependent relationship between ANA and triglycerides, as no association was observed between ANA status and serum triglyceride levels ≥200 mg/dL. Given that our study was based on secondary analysis of NHANES data, we are not able to identify mechanisms to explain these effects, or rule out residual confounding factors. Our analysis was additionally limited by a relatively small sample size (*n* = 7 for men with triglyceride levels within 150 to <200 mg/dL). Thus, further investigation is warranted to confirm and explore these observations. Additionally, while regulation of systemic lipolysis varies between men and women (89), the mechanisms underlying sex-specific associations between ANA and serum triglycerides are unclear, and warrant further study.

In line with the associations between dyslipidemias and autoimmune disease risk (34–38), lipid-lowering statins have been shown to exert protective and anti-inflammatory effects within the context of autoimmunity (5, 39–41, 90, 91). Statins have been shown to improve clinical outcomes in SLE and RA patients, and have been reported to reduce the risk of developing RA (39–41). Interestingly, we observed a sex-specific effect in evaluating the relationship between statin use and ANA status, in that women who reported taking statins had significantly lower odds of being ANA+, whereas no significant association between statin use and ANA status was observed in men. While the mechanism underlying this observation is unclear, it has been reported that statins may have variable efficacy in men vs. women in regards to cardiovascular disease risk (92, 93). Women have also reported greater side effects in taking statins as compared to men, including muscle symptoms indicative of statin-induced myopathy (94, 95). These findings warrant further investigation as to whether statins have greater immunomodulatory properties in women, which would have significant implications for clinical care and autoimmune disease treatment.

In conclusion, the findings from this study provide insight into the relationship between lipid metabolism and autoimmunity by elucidating the limited, albeit sex-specific utility of routine clinical serum lipid levels to predict ANA status at the population level. Most importantly, our study identifies a sex-specific and protective role for statins in predicting ANA status in women, who are most likely to be diagnosed with autoimmune conditions and experience statin intolerance (11, 94). Given the strength of preclinical evidence that points to a significant role for lipoproteins in modulating leukocyte activity and autoimmune outcomes, further clinical and population-based studies to evaluate the utility of serum lipids or other routine lipid biomarkers in predicting autoimmune outcomes, as well as elucidate potential therapeutic opportunities and limitations of statins in treating rheumatic diseases, is warranted.

## Data Availability Statement

Publicly available datasets were analyzed in this study. This data can be found here: https://www.cdc.gov/nchs/nhanes/.

## Ethics Statement

The studies involving human participants were reviewed and approved by the National Center for Health Statistics Research Ethics Review Board. The participants provided their written informed consent.

## Author Contributions

CA: conceptualization, data analysis, and drafted the manuscript. TV: statistical methodology and analysis, review, and contribution of writing to the manuscript. CA and TV: data interpretation. Both authors approve of the final manuscript.

## Funding

Open access publication fees were supported by University of Connecticut start-up funds to CA.

## Conflict of Interest

The authors declare that the research was conducted in the absence of any commercial or financial relationships that could be construed as a potential conflict of interest.

## Publisher's Note

All claims expressed in this article are solely those of the authors and do not necessarily represent those of their affiliated organizations, or those of the publisher, the editors and the reviewers. Any product that may be evaluated in this article, or claim that may be made by its manufacturer, is not guaranteed or endorsed by the publisher.

## Abbreviations

ANA: antinuclear antibodies
BMI: body mass index
ERα: Estrogen receptor α
HDL-C: HDL-cholesterol
ID HPLC-APCI MS/MS: isotope dilution-high performance liquid chromatography/atmospheric pressure chemical ionization tandem mass spectrometry
IgG: immunoglobulin G
LDL-C: LDL-cholesterol
NHANES: National Health and Nutrition Examination Survey
RA: rheumatoid arthritis
SLE: systemic lupus erythematous
T_H_1: T helper 1 cells
T_H_2: T helper 1 cells
T_H_17: T helper 1 cells
T_reg_: T regulatory cells.
